# Assessment of Microvascular Function in Angina Pectoris by Angiography-Based Index of Microcirculation Resistance: A Meta-Analysis

**DOI:** 10.31083/RCM25764

**Published:** 2025-08-25

**Authors:** Wei Wen, Yi Chi, Mingwang Liu, Beili Xie, Mengjie Gao, Lulian Jiang, Yiqing Zhang, Keji Chen, Fuhai Zhao

**Affiliations:** ^1^Cardiovascular Department, Xiyuan Hospital of China Academy of Chinese Medical Sciences, 100091 Beijing, China; ^2^Geriatrics Department, The People's Hospital Medical Group of Xiangzhou, 519000 Zhuhai, Guangdong, China; ^3^National Clinical Research for Chinese Medicine Cardiology, 100091 Beijing, China

**Keywords:** angina pectoris, coronary artery disease, angiography-based index of microcirculation resistance, index of microcirculation resistance, coronary microvascular dysfunction

## Abstract

**Background:**

While the invasive index of microcirculation resistance (IMR) remains the gold standard for diagnosing coronary microvascular dysfunction (CMD), its clinical adoption is limited by procedural complexity and cost. Angiography-based IMR (Angio-IMR), a computational angiography-based method, offers a promising alternative. This study evaluates the diagnostic efficacy of Angio-IMR for CMD detection in angina pectoris (AP).

**Methods:**

A comprehensive literature search was conducted across PubMed, Embase, Scopus, and the Cochrane Library to identify studies assessing Angio-IMR's diagnostic performance for CMD in AP populations. Primary outcomes included pooled sensitivity, specificity, positive predictive value (PPV), negative predictive value (NPV), and area under the receiver operating characteristic (ROC) curve (AUC).

**Results:**

11 studies involving 927 patients were included. Angio-IMR demonstrated robust diagnostic performance: sensitivity 86% (95% CI: 0.83–0.90), specificity 90% (95% CI: 0.87–0.92), PPV 82% (95% CI: 0.78–0.86), NPV 91% (95% CI: 0.88–0.94), and AUC 0.91 (95% CI: 0.89–0.94), with low heterogeneity. Subgroup analyses revealed no significant differences in diagnostic accuracy between obstructive (stenosis ≥50%) and non-obstructive coronary artery disease. Hyperemic Angio-IMR measurements (adenosine-induced) showed superior sensitivity (89% vs. 86%) and specificity (94% vs. 91%) compared to resting-state assessments by AccuFFR system. Additionally, the sensitivity (88% vs. 82%), specificity (92% vs. 86%), PPV (82% vs. 78%) and NPV (91% vs. 88%) calculated based on AccuFFR were higher than that of quantitative flow ratio (QFR).

**Conclusions:**

Angio-IMR is a reliable, non-invasive tool for CMD identification in angina patients, particularly under hyperemic conditions. Its diagnostic consistency across stenosis severity subgroups supports broad clinical applicability.

## 1. Introduction

Research indicates that ischemic heart disease (IHD) is a critical cause of cardiac death [1, 2]. The incidence of myocardial infarction in women is closely associated with the progressive rise in fatal IHD rates with advancing age [3]. As of 2018, the IHD mortality rate among non-Hispanic white women reached 64.9% per 100,000 population [3]. A U.S. study revealed that since 2000, there has been minimal improvement in IHD mortality among young women [4], which may be linked to insufficient risk communication by physicians. A survey showed that only 21% of women were informed about the potential adverse outcomes of IHD [5]. Although prior studies have exhaustively analyzed the poor prognosis of IHD, it remains a syndrome encompassing complex pathophysiology [6, 7, 8], with its conceptual scope extending beyond myocardial ischemia caused by atherosclerosis. Notably, the ORBITA (Optimal Medical Therapy of Angioplasty in Stable Angina) [9] and ISCHEMIA (International Study of Comparative Health Effectiveness with Medical and Invasive Approaches) [10] trials have challenged the traditional coronary stenosis-centric therapeutic paradigm for IHD. Furthermore, the SCOT-HEART (Scottish Computed Tomography of the Heart) study [11] demonstrated that most patients with coronary heart disease (CHD) lack epicardial stenosis, suggesting that angina symptoms and ischemic manifestations in non-obstructive CHD may stem from impaired microvascular regulation. Consequently, IHD caused by coronary microvascular dysfunction (CMD) has emerged as a pivotal factor in evaluating coronary revascularization and prognosis, making coronary microvascular disease a pressing public health challenge.

CMD is characterized by functional and structural abnormalities in the microvasculature (non-atherosclerotic stenosis) that disrupt coronary blood flow regulation, manifesting as enhanced microvascular constriction, impaired endothelium-dependent/independent vasodilation, and elevated microcirculation resistance [12, 13]. Diagnosis of CMD relies on imaging and functional assessments, with the pressure wire-derived index of microcirculation resistance (IMR) currently serving as a key tool for evaluating coronary microvascular disease (CMVD) [14, 15, 16, 17]. However, this invasive approach requires pharmacologically induced maximal coronary hyperemia to obtain measurements, often causing adverse effects such as chest tightness and bradycardia. Recent research has focused on angiography-based IMR (Angio-IMR), a non-invasive method that indirectly assesses coronary functional parameters without additional procedures. This study aims to evaluate the diagnostic efficiency of Angio-IMR for identifying CMD in populations suffering from angina pectoris.

## 2. Materials and Methods

### 2.1 Literature Search Strategy

We performed a systematic literature search across PubMed (https://pubmed.ncbi.nlm.nih.gov/), Embase (http://www.embase.com), Scopus (https://www.scopus.com), and the Cochrane Library (http://www.thecochranelibrary.com). To ensure comprehensive coverage, no population restrictions were imposed, and all search terms related to Angio-IMR and its variants (including AMR, CaIMR, and AccuIMR) were systematically retrieved. The complete search strategy is detailed in **Supplementary Data**.

### 2.2 Inclusion and Exclusion Criteria

Two investigators (WW and YC) independently screened studies through a two-phase process: an initial review of titles and abstracts followed by full-text review. Discrepancies in eligibility assessment were resolved through adjudication by a third researcher (FHZ). Studies were retained if they were in accord with the inclusion criteria: (i) IMR was quantified using a pressure guide wire; (ii) Participants had angina pectoris, including chronic coronary syndrome (CCS), stable angina, or unstable angina; (iii) The study reported diagnostic performance metrics (e.g., sensitivity, specificity) of angiography-derived IMR (Angio-IMR) for detecting CMD. The main exclusion criteria included: (i) Absence of extractable diagnostic metrics including sensitivity, specificity, positive predictive value (PPV), and negative predictive value (NPV); (ii) Lack of Angio-IMR reporting; (iii) IMR measurements in patients with cardiomyopathy, valvular heart disease, coronary artery bypass grafts (CABG), or cardiac transplants; (iv) Duplicate literature, animal experiments, or non-research articles.

### 2.3 Data Extraction and Literature Quality Assessment

Two investigators independently conducted data extraction with subsequent cross-validation to ensure accuracy. The collected variables comprised author information, publication year, research design, and baseline demographic characteristics. For methodological quality evaluation, the Quality Assessment of Diagnostic Accuracy Studies-2 (QUADAS-2) tool [18] was employed to assess four critical dimensions: patient selection criteria, index test methodology, reference standard validity, and temporal consistency in testing procedures [18].

### 2.4 Statistical Analyses

Diagnostic accuracy metrics—including sensitivity, specificity, PPV and NPV, and their 95% confidence intervals (95% CI)—were extracted from the included studies. Statistical analyses were performed using Stata 15.0 (StataCorp LLC, College Station, TX, USA) to calculate pooled estimates of sensitivity, specificity, PPV, NPV, and the area under the receiver operating characteristic (ROC) curve (AUC). Study quality was appraised with Review Manager 5.4 (The Cochrane Collaboration, Oxford, UK). A random-effects model was employed for meta-analysis, with forest plots generated to visualize effect sizes across studies. Heterogeneity was assessed using Higgins’ *I*^2^ statistic (α = 0.05), interpreted as follows: *I*^2^ < 50% indicates the low heterogeneity, *I*^2^ ≥ 50% indicates the higher heterogeneity, and *I*^2^ ≥ 75% indicates high heterogeneity, necessitating subgroup analyses to identify potential sources [19].

## 3. Results

### 3.1 Results of the Literature Search

**Fig. 1.**
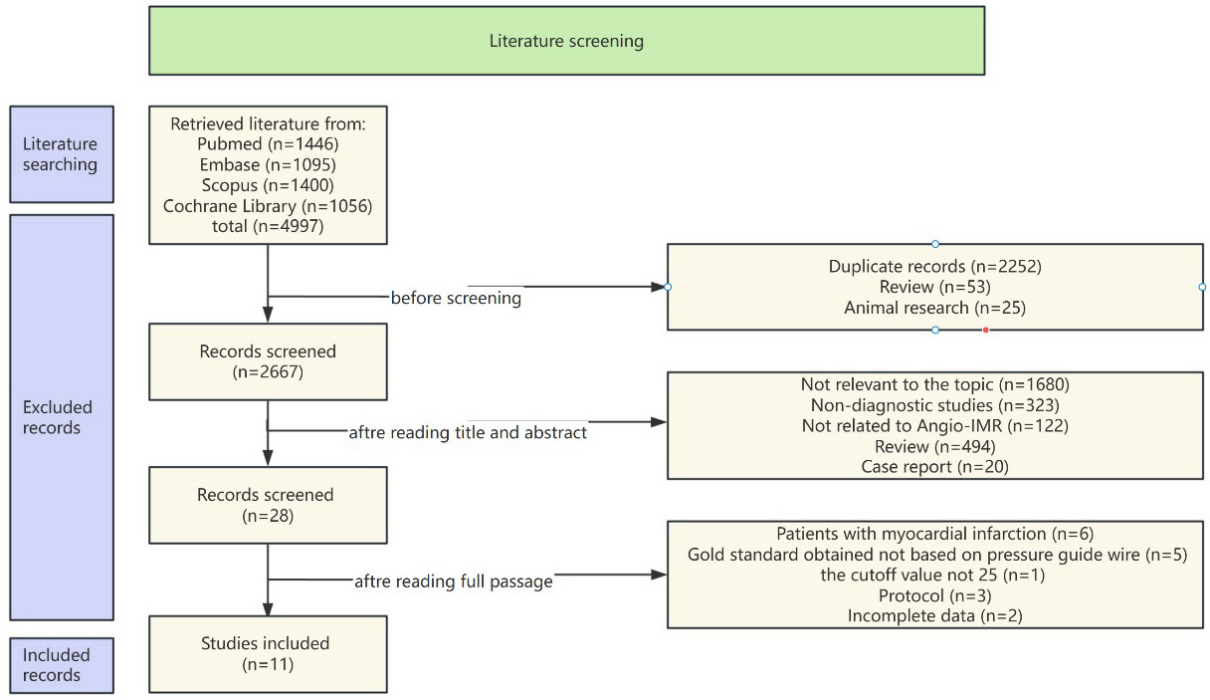
**Flow chart**. Angio-IMR, angiography-based instantaneous wave-free ratio.

The systematic search protocol identified 4997 potentially relevant records across four biomedical databases: PubMed (n = 1446), Embase (n = 1095), Scopus (n = 1400), and Cochrane Library (n = 1056). Following rigorous screening procedures, 11 eligible studies [17, 20, 21, 22, 23, 24, 25, 26, 27, 28, 29] met the inclusion criteria, with the complete selection workflow visually delineated in Fig. 1.

The study included first author, year, country, number of cases in the included studies, baseline characteristics, Angio-IMR cut-off value, study population, and study design, as detailed in Table 1 (Ref. [17, 20, 21, 22, 23, 24, 25, 26, 27, 28, 29]).

**Table 1.** **Basic table**.

First Author	Year	Country	Study design	Disease	Number of patients	AGE (years)	Male (%)	Number of vessels	Cutoff value	Angiography‐based FFR
Zhongjue Qiu [20]	2024	China	single‐center	CCS	75	54.30 ± 12.99	36 (48%)	79	2.6 mmHg·s/cm	QFR
Beibei Gao [21]	2024	China	single‐center	CCS	66	67.74 ± 9.38	37 (56%)	103	2.66 mmHg·s/cm	QFR
Chenguang Li [22]	2023	China	single‐center	CCS	101	61 ± 10	78 (77%)	101	unknown	AccuFFR
Yongzhen Fan [23]	2023	China	single‐center	CCS	61	unknown	unknown	unknown	unknown	AccuFFR
Dong Huang [17]	2023	China	multi-center	INOCA	116	62.9 ± 8	64 (55%)	113	unknown	caFFR
Jun Jiang [24]	2022	China	multicenter	CCS	203	64	140 (69%)	203	unknown	AccuFFR
Hernan Mejia-Renteria [25]	2021	UK	multicenter	CCS	104	64.2 ± 11.1	79 (76%)	115	unknown	QFR
Matteo Tebaldi [26]	2020	Italy	single‐center	CCS	44	70	36 (82%)	44	25 U	QFR
Hu Ai [27]	2020	China	multicenter	INOCA	56	61.9 ± 9.2	30 (54%)	57	25 U	AccuFFR
Roberto Scarsini [28]	2021	UK	single‐center	CCS	36	67	24 (67%)	52	25 U	QFR
Yongzhen Fan [29]	2025	China	single‐center	INOCA	65	64 ± 10.4	unknown	unknown	25 U	unknown

CCS, chronic coronary syndrome; INOCA, ischemia with non-obstructive coronary arteries; UK, The United Kingdom of Great Britain and Northern Ireland; IMR, index of microcirculation resistance; FFR, fractional flow reserve; U, unit; QFR, quantitative flow ratio; AccuFFR, accelerated fractional flow reserve; CaFFR, coronary angiography-derived fractional flow reserve.

### 3.2 Evaluation of the Quality of Literature

**Fig. 2.**
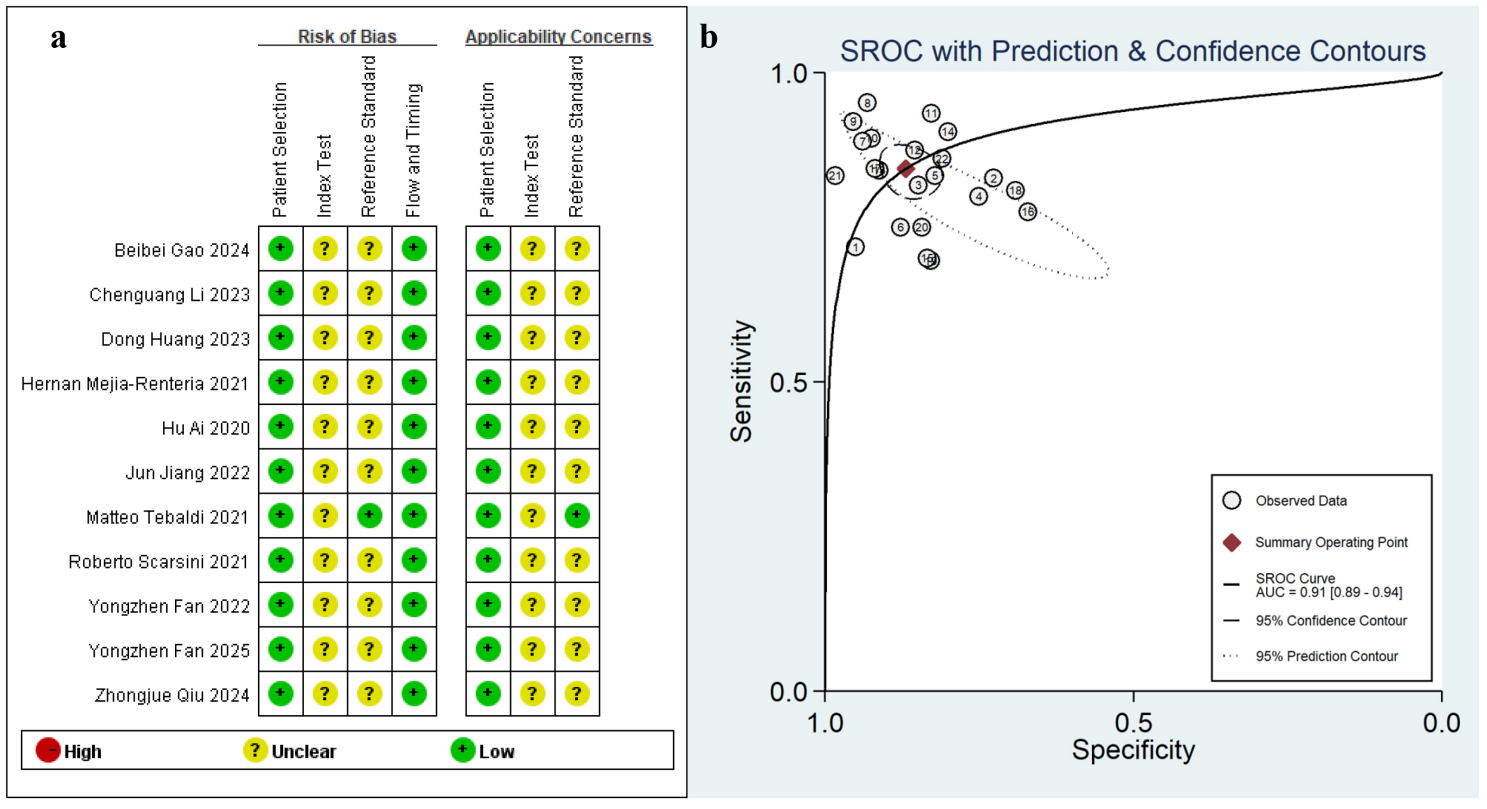
**Cumulative traffic light plots and weighted bar plots of the risk of bias (a), the summary receiver operating characteristic curve (SROC) (b)**. AUC, area under the curve.

Methodological appraisal conducted via QUADAS-2 (Fig. 2) revealed inherent validity concerns. All studies prospectively enrolled consecutive patients with rigorously defined exclusion criteria; thus, none employed a case-control design. However, potential bias may arise from the lack of predefined diagnostic cutoff value for Angio-IMR in these prospective studies. Notably, most trials adopted a blinded design to minimize observer bias, and the diagnostic criteria for CMD based on the gold standard (IMR) followed consensus-derived cutoffs [30].

### 3.3 Diagnostic Accuracy of Angio-IMR

#### 3.3.1 Pooled Overall Results

**Fig. 3.**
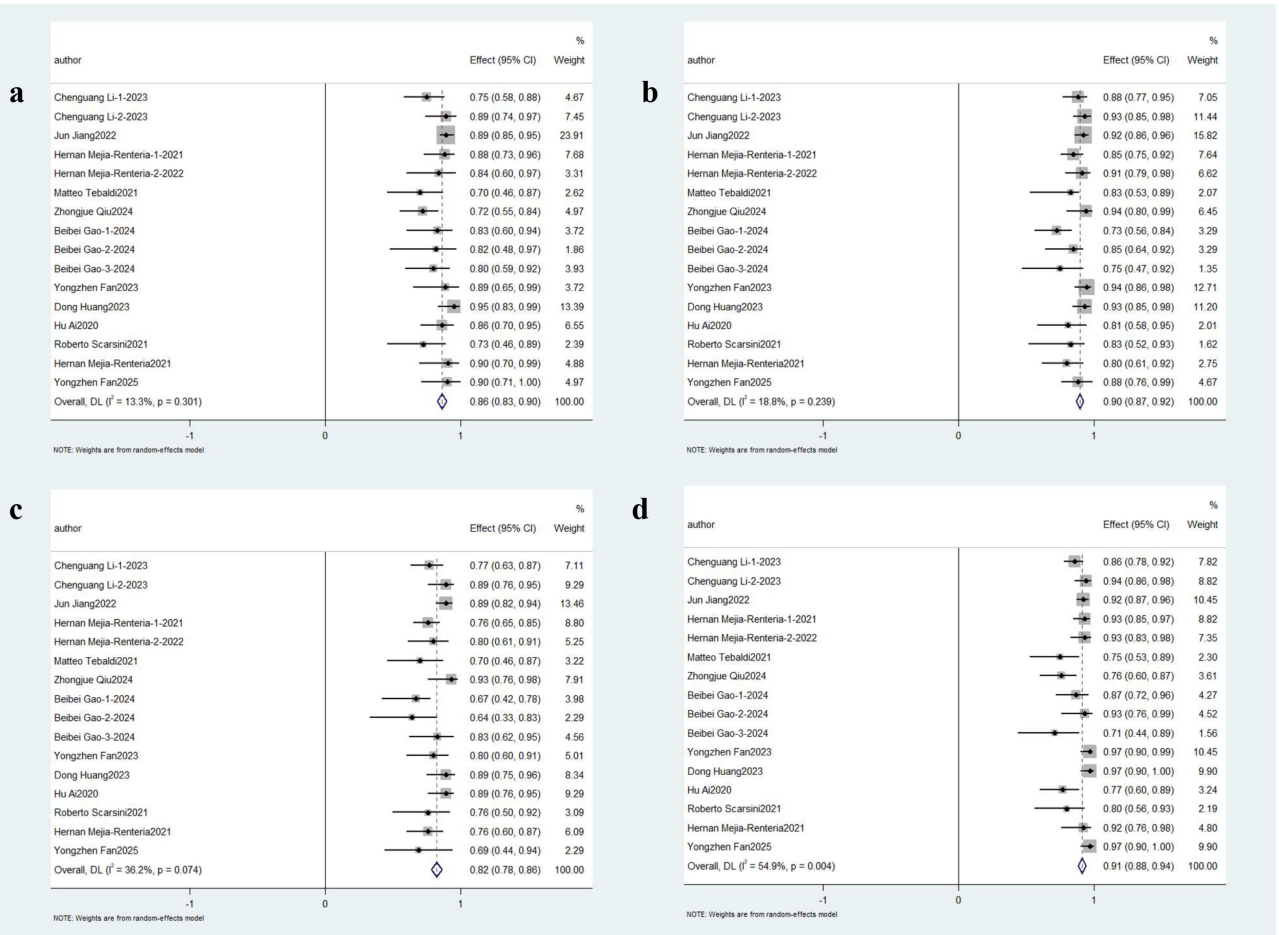
**Pooled Overall Results. Forest plots showing the pooled sensitivity (a), specificity (b), positive predictive value (c) and negative predictive value (d)**.

11 eligible studies involving 927 participants were analyzed in this meta-analysis. Diagnostic performance evaluation demonstrated Angio-IMR exhibited 86% sensitivity (95% CI: 0.83–0.90; *I*^2^ = 13.3%, *p* < 0.01) and 90% specificity (95% CI: 0.87–0.92; *I*^2^ = 18.8%, *p* < 0.01). The PPV reached 82% (95% CI: 0.78–0.86; *I*^2^ = 36.2%, *p* < 0.01), while the NPV was notably higher at 91% (95% CI: 0.88–0.94; *I*^2^ = 54.9%, *p* < 0.01), as shown in Fig. 3. Notably, the comprehensive diagnostic accuracy reflected by the AUC was 0.91 (95% CI: 0.89–0.94), as illustrated in Fig. 2.

#### 3.3.2 Subgroup Analysis

3.3.2.1 Obstructive CAD and non-obstructive CAD

**Fig. 4.**
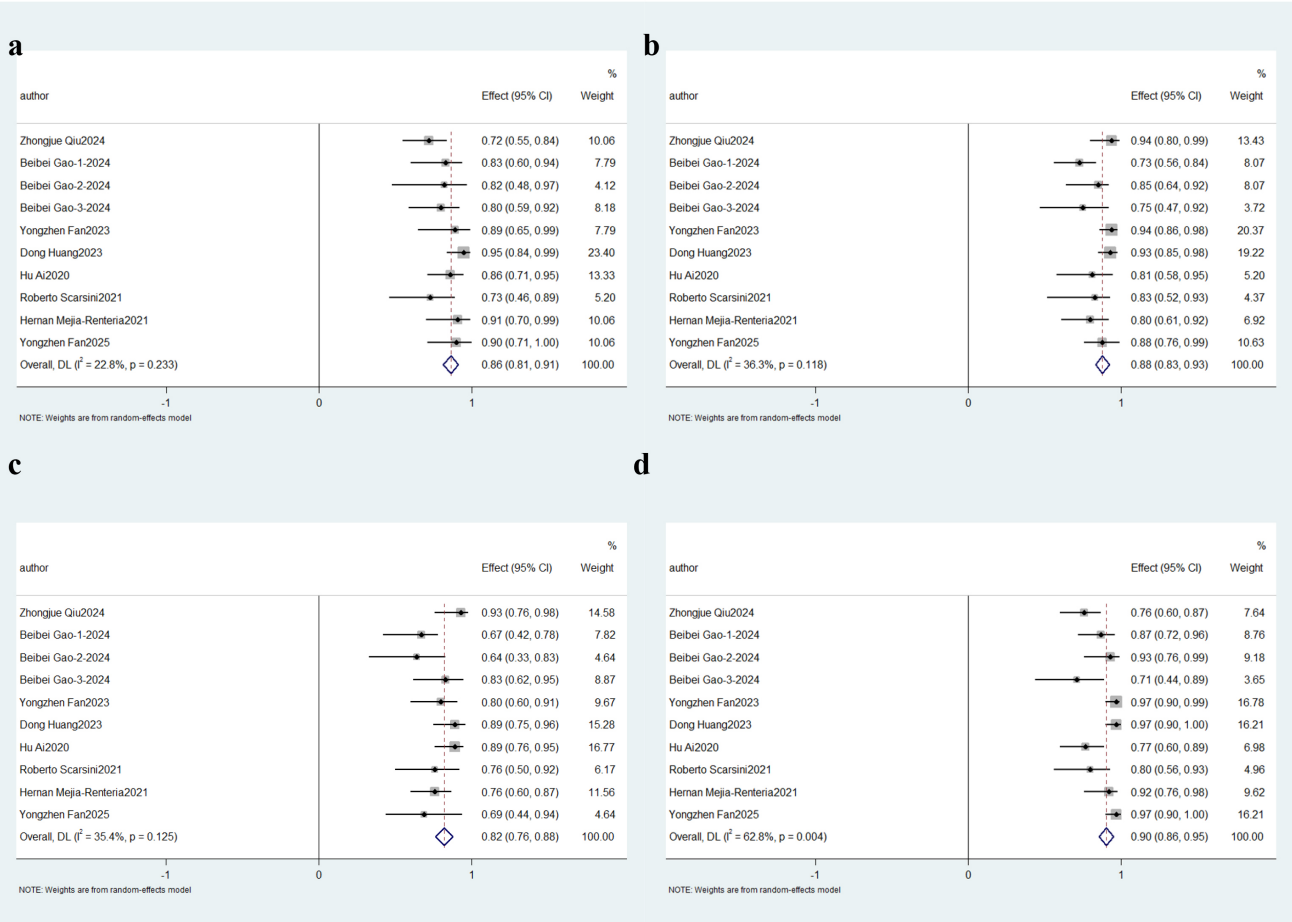
**Description of cumulative results in non-obstructive CAD**. Forest plots showing the pooled sensitivity (a), specificity (b), positive predictive value (c) and negative predictive value (d).

Subgroup analyses were stratified based on clinical characteristics. Three studies focused on ischemia with non-obstructive coronary arteries (INOCA) cohorts, while five [17, 20, 21, 23, 28] evaluated Angio-IMR in target vessels post-percutaneous coronary intervention (PCI), collectively representing eight studies involving patients with non-obstructive coronary artery disease. The aggregate sensitivity remained robust at 86% (95% CI: 0.81–0.91; *p* < 0.01, *I*^2^ = 22.8%), with specificity reaching 88% (95% CI: 0.83–0.93; *I*^2^ = 36.3%, *p* < 0.01). Diagnostic precision analysis revealed an 82% PPV (95% CI: 0.76–0.88; *I*^2^ = 35.4%, *p* < 0.01) and a significantly higher NPV of 90% (95% CI: 0.86–0.95; *I*^2^ = 62.8%, *p* < 0.01), as detailed in Fig. 4.

**Fig. 5.**
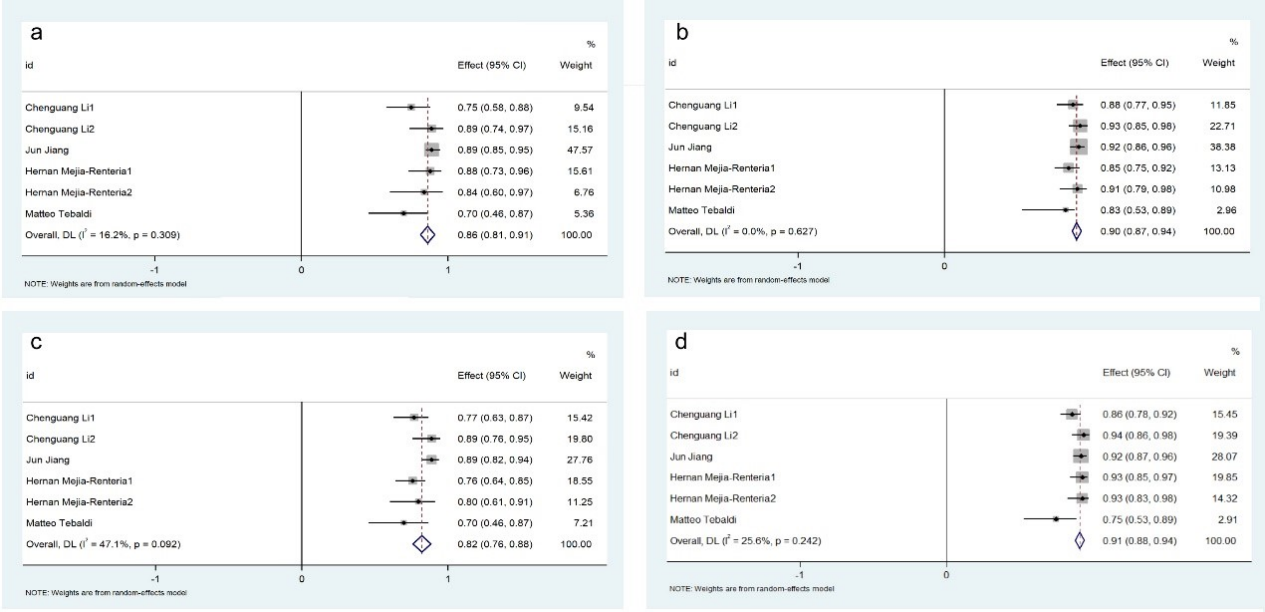
**Description of cumulative results in obstructive CAD**. Forest plots showing the pooled sensitivity (a), specificity (b), positive predictive value (c) and negative predictive value (d).

Four studies specifically investigated obstructive CAD populations, defined by coronary stenosis ≥50% or FFR <0.8. The combined diagnostic performance metrics demonstrated 86% sensitivity (95% CI: 0.81–0.91; *p* < 0.01, *I*^2^ = 16.2%) and 90% specificity (95% CI: 0.87–0.94; *I*^2^ = 0.0%, *p* < 0.01). Predictive validity analysis revealed an 82% PPV (95% CI: 0.76–0.88; *I*^2^ = 47.1%, *p* < 0.01) alongside a clinically significant NPV of 91% (95% CI: 0.88–0.94; *I*^2^ = 25.6%, *p* < 0.01), as visualized in Fig. 5.

3.3.2.2 Angiography-based fractional flow reserve (FFR)

**Fig. 6.**
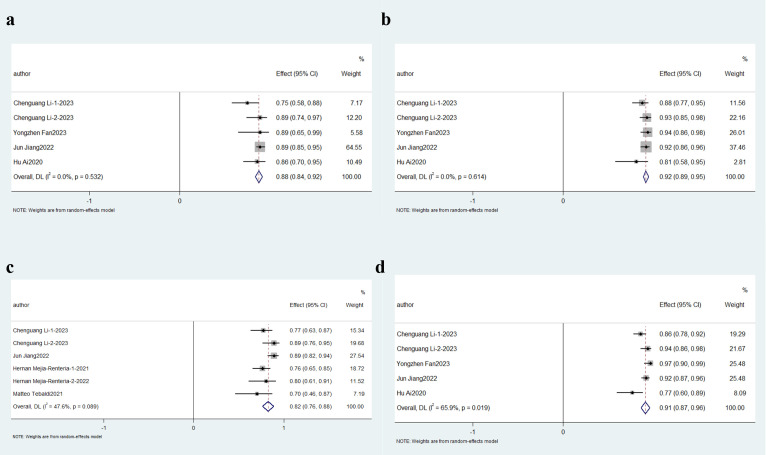
**Description of cumulative results based on AccuFFR**. Forest plots showing the pooled sensitivity (a), specificity (b), positive predictive value (c) and negative predictive value (d).

Four independent studies utilizing the Accelerated Fractional Flow Reserve (AccuFFR) computational platform for angio-IMR quantification were analyzed. This subgroup demonstrated exceptional diagnostic consistency, with aggregated sensitivity and specificity reaching 88% (95% CI: 0.84–0.92; *p <*0.01, *I*^2^ = 0.0%) and 92% (95% CI: 0.89–0.95; *I*^2^ = 0.0%, *p <*0.01) respectively. The predictive capacity analysis yielded an 82% PPV (95% CI: 0.76–0.88; *I*^2^ = 47.6%, *p* < 0.01), contrasted with a superior NPV of 91% (95% CI: 0.87–0.96; *I*^2^ = 65.9%, *p <*0.01). These metrics collectively indicate robust diagnostic accuracy, as depicted in Fig. 6.

**Fig. 7.**
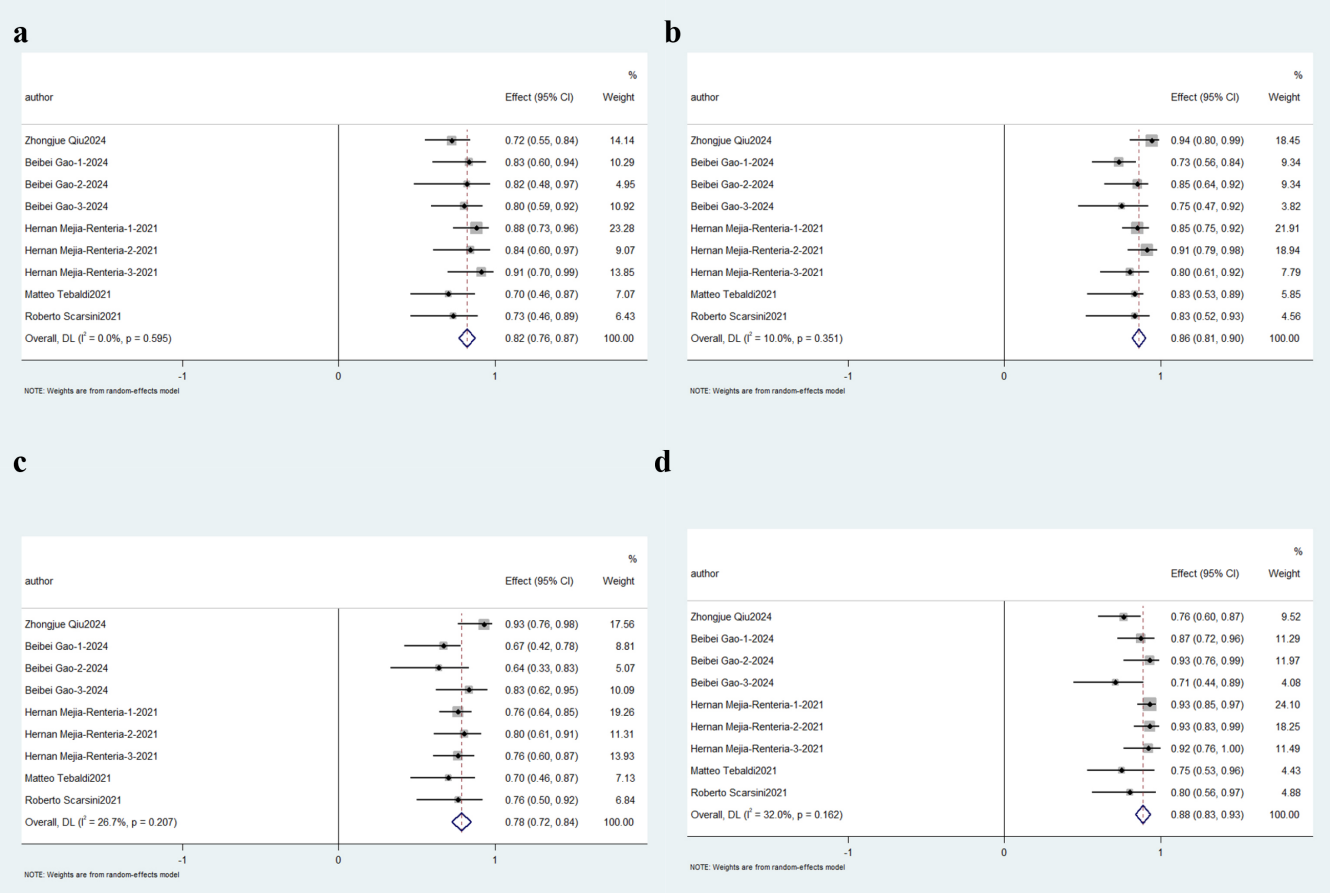
**Description of cumulative results in based on QFR**. Forest plots showing the pooled sensitivity (a), specificity (b), positive predictive value (PPV)(c) and negative predictive value (NPV)(d).

Five investigations employing quantitative flow ratio (QFR) assessment methodologies were included in this sub-analysis. Diagnostic accuracy metrics demonstrated 82% aggregate sensitivity (95% CI: 0.76–0.87; *p* < 0.01, *I*^2^ = 0.0%) with corresponding specificity of 86% (95% CI: 0.81–0.90; *I*^2^ = 10.0%. *p* < 0.01). The clinical utility profile revealed 78% positive predictive capacity (95% CI: 0.72–0.84; *I*^2^ = 26.7%, *p* < 0.01), while negative predictive performance achieved 88% accuracy (95% CI: 0.83–0.93; *I*^2^ = 32.0%, *p* < 0.01). These stratified outcomes are comprehensively visualized in Fig. 7.

3.3.2.3 The impact of vasodilators (adenosine) based on AccuFFRangio system

**Fig. 8.**
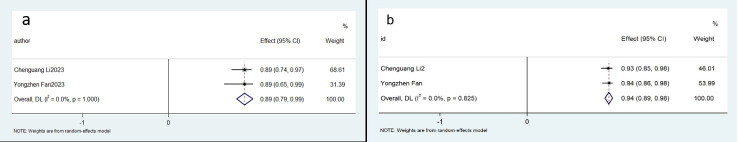
**Description of cumulative results using the AccuFFRangio software in the hyperemic state**. Forest plots showing the pooled sensitivity (a) and specificity (b).

**Fig. 9.**
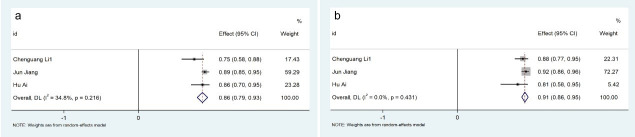
**Description of cumulative results using the AccuFFRangio software in the rest state**. Forest plots showing the pooled sensitivity (a) and specificity (b).

In-depth physiological state stratification revealed distinct diagnostic performance profiles. Within the AccuFFRangio platform, hyperemic state assessments (n = 2 studies) achieved 89% aggregate diagnostic sensitivity (95% CI: 0.79–0.99; *p* < 0.01, *I*^2^ = 0.0%) with 94% specificity (95% CI: 0.89–0.98; *I*^2^ = 0.0%, *p* < 0.01), as detailed in Fig. 8. Contrastingly, resting state evaluations (n = 3 studies) demonstrated 86% sensitivity (95% CI: 0.79–0.93; *p* < 0.01, *I*^2^ = 34.8%) and 91% specificity (95% CI: 0.86–0.95; *I*^2^ = 0.0%, *p* < 0.01) under identical computational framework conditions (Fig. 9).

## 4. Discussion

Our study demonstrates that Angio-IMR exhibits high diagnostic concordance with invasive pressure wire-derived IMR for detecting CMD. Pooled estimates revealed clinically robust performance indicators, involving sensitivity, specificity, PPV, and NPV. The superior discriminative capacity of Angio-IMR was further evidenced by AUC >0.9, supporting its utility as a non-invasive alternative to guide CMD diagnosis in clinical practice.

Patients with angina attributable to CMD constitute a clinically significant subset of those with chronic coronary syndrome (CCS). Although CMD is traditionally classified as a non-atherosclerotic disorder, the Women’s Ischemia Syndrome Evaluation (WISE) substudy employing intravascular ultrasound (IVUS) identified female patients with non-obstructive CAD and revealed that CMD-associated cardiovascular risk correlates strongly with atherosclerosis-related risk factors [31, 32, 33]. Current epidemiological data estimate the prevalence of coronary microvascular disease (CMVD) at 40–60% [34, 35], though heterogeneity persists due to inconsistent diagnostic criteria for CMD. Furthermore, the reliance on invasive pressure wire-derived IMR has limited the detection rate of CMD in clinical practice. These limitations have driven the development of Angio-IMR, a non-invasive computational framework rooted in the hemodynamic principles of invasive IMR. Specifically, invasive IMR is characterized as the product of distal coronary pressure (Pd) and mean transit time (Tmn) when reaching the maximal hyperemic state [36]. Current Angio-IMR algorithms primarily derive from this foundational formula [2, 17, 20]:

(1) $$A\,M\,R=\frac{P\,d}{V\,e\,l\,o\,c\,i\,t\,y_{h\,y\,p}}=\frac{P\,a\times Q\,F\,R}{V\,e\,l\,o\,c\,i\,t\,y_{h\,y\,p}}$$

(2) $$A\,c\,c\,u\,I\,M\,R=\frac{P\,a\times A\,c\,c\,u\,F\,F\,R\,a\,n\,g\,i\,o\times L}{V\,e\,l\,o\,c\,i\,t\,y_{h\,y\,p}}$$

(3) $$c\,a\,I\,M\,R=\frac{P\,d\times L}{K*V\,e\,l\,o\,c\,i\,t\,y_{h\,y\,p}}$$

Pd: distal coronary pressure, QFR: quantitative flow ratio, Pa: proximal coronary pressure, L: length of blood vessels, Velocity_hyp_: flow velocity in the hyperemic status.

The derivation of angiography-based IMR fundamentally involves simulating distal coronary pressure (Pd) via FFR calculations [37], followed by multiplying Pd by aortic pressure (Pa) and contrast transit time (Tmn). This approach hinges on angiography-derived FFR computations, with microvascular resistance (MR) subsequently quantified through computational fluid dynamics (CFD). Large-scale trials have demonstrated non-inferiority of angiography-based FFR-guided percutaneous coronary intervention (PCI) compared to wire-based FFR strategies [38], validating its equivalence in assessing stenotic epicardial vessel function. However, there is limited evidence regarding the correlation between pressure wire-based IMR and Angio-IMR. Furthermore, prior meta-analyses exhibited significant methodological bias due to heterogeneous cohorts (e.g., combining CCS and acute coronary syndrome populations) [39] and inconsistent diagnostic thresholds for the gold-standard IMR across studies. To address these limitations, this meta-analysis exclusively focused on angina populations (excluding myocardial infarction) to minimize confounding variables.

Based on angiographic stenosis severity, enrolled patients were stratified into two cohorts: obstructive CAD (stenosis ≥50%) and non-obstructive CAD. Subgroup analysis revealed comparable diagnostic efficiency of Angio-IMR in both groups, with insignificant differences in sensitivity or specificity, suggesting that coronary stenosis severity does not compromise Angio-IMR’s ability to identify CMD. This finding aligns with evidence from Scarsini *et al*. [28], who demonstrated strong correlation between Angio-IMR and invasive IMR across infarct-related arteries (IRA), non-IRA, and diverse clinical presentations (STEMI, NSTEMI, and CCS). Furthermore, subgroup comparisons of angiography-derived FFR computational platforms (quantitative FFR [QFR] vs. AccuFFR) indicated superior diagnostic accuracy for Accu-IMR derived from AccuFFR.

Given the inclusion of four studies [22, 24, 25, 26] utilizing the AccuFFRangio system to quantify Angio-IMR, we performed additional subgroup analyses. These revealed that hyperemia-induced Angio-IMR measurements (achieved via adenosine-mediated vasodilation) exhibit superior reliability compared to resting-state assessments. This is consistent with the findings of Scarsini *et al*. [28], who revealed no significant correlation between non-hyperemic Angio-IMR and invasive IMR in CCS cohorts. Collectively, these observations underscore the necessity of hyperemic conditions for optimizing Angio-IMR’s diagnostic utility in CMD.

While the limited number of included studies and heterogeneity in computational platforms introduce potential confounding biases, subgroup analyses reinforced the robustness of the primary findings. The meta-analysis conclusively demonstrates that Angio-IMR achieves high diagnostic performance (AUC: 0.91, 95% CI: 0.89–0.94), indicating its clinical applicability as a non-invasive alternative.

## 5. Conclusions

This comprehensive meta-analytical synthesis establishes angio-IMR as a diagnostically robust modality, demonstrating superior discriminative capacity for detecting CMD in angina pectoris cohorts. The concordance with invasive wire-based IMR measurements collectively confirms its clinical validity, thereby positioning this modality as a viable non-invasive surrogate for traditional intracoronary physiological assessment.

## Availability of Data and Materials

The original data for this study is available from the corresponding author.

## Supplementary Material

Supplementary material associated with this article can be found, in the online version, at https://doi.org/10.31083/RCM25764.
