# Supplementary material for: Assessment of Microvascular Function in Angina Pectoris by Angiography-Based Index of Microcirculation Resistance: A Meta-Analysis
Source: Rev Cardiovasc Med. 2025 Aug 25;26(8):25764. doi: 10.31083/RCM25764 (PMC12415731; doi:10.31083/RCM25764)
Supplement: Supplementary file 1 [file 2153-8174-26-8-25764-s1.zip › Supplementary Material.docx]

**Supplementary Table 1** Search strategies

| **Databases** | **Search strategies** |
| --- | --- |
| PubMed | ((((((((((((((((((((((((((((("angiography-derived index of microcirculatory resistance"[Title/Abstract]) OR ("angiography-derived coronary microcirculatory resistance index"[Title/Abstract])) OR ("angiography-derived index of microvascular resistance"[Title/Abstract])) OR ("angio- index of coronary microcirculatory resistance"[Title/Abstract])) OR ("angio- index of microcirculatory resistance"[Title/Abstract])) OR ("angio- index of microvascular resistance"[Title/Abstract])) OR ("angio-IMR"[Title/Abstract])) OR ("angiography-derived IMR"[Title/Abstract])) OR ("Angio-Based Index of Microcirculatory Resistance"[Title/Abstract])) OR ("Angio-Based Index of microvascular Resistance"[Title/Abstract])) OR ("A-IMR"[Title/Abstract])) OR ("Angio-IMR"[Title/Abstract])) OR ("angiographic microvascular resistance"[Title/Abstract])) OR ("angiographic microcirculatory resistance"[Title/Abstract])) OR ("Angiography-based index of microcirculatory resistance"[Title/Abstract])) OR ("AccuIMR"[Title/Abstract])) OR ("coronary angiography-derived index of microcirculatory resistance"[Title/Abstract])) OR ("coronary angiography-derived index of microvascular resistance"[Title/Abstract])) OR ("CaIMR"[Title/Abstract])) OR ("Index of Microvascular resistance"[Title/Abstract])) OR ("index of microcirculatory resistance"[Title/Abstract])) OR ("IMRangio"[Title/Abstract])) OR ("Microcirculatory Resistance Index Based on Imaging"[Title/Abstract])) OR ("Microvascular Resistance Index Based on Imaging"[Title/Abstract])) OR ("Quantitative flow ratio derived index of microcirculatory resistance"[Title/Abstract])) OR ("Quantitative Microvascular resistance"[Title/Abstract])) OR ("Quantitative microcirculatory resistance"[Title/Abstract])) OR ("quantitative MR"[Title/Abstract])) OR ("quantitative index of Microvascular resistance"[Title/Abstract])) OR ("quantitative index of microcirculatory resistance"[Title/Abstract]) |
| Cochrane Library | (angiography-derived index of microcirculatory resistance):ti,ab,kw OR (angiography-derived coronary microcirculatory resistance index):ti,ab,kw OR (angiography-derived index of microvascular resistance):ti,ab,kw OR (angio-index of coronary microcirculatory resistance):ti,ab,kw OR (angio-index of microcirculatory resistance):ti,ab,kw OR (angio-index of microvascular resistance):ti,ab,kw OR (angio-IMR):ti,ab,kw OR (angiography-derived IMR):ti,ab,kw OR (Angio-Based Index of Microcirculatory Resistance):ti,ab,kw OR (Angio-Based Index of microvascular Resistance):ti,ab,kw OR (A-IMR):ti,ab,kw OR (Angio-IMR):ti,ab,kw OR (angiographic microvascular resistance):ti,ab,kw OR (angiographic microcirculatory resistance):ti,ab,kw OR (Angiography-based index of microcirculatory resistance):ti,ab,kw OR (AccuIMR):ti,ab,kw OR (coronary angiography-derived index of microcirculatory resistance):ti,ab,kw OR (coronary angiography-derived index of microvascular resistance):ti,ab,kw OR (CaIMR):ti,ab,kw OR (Index of Microvascular resistance):ti,ab,kw OR (index of microcirculatory resistance):ti,ab,kw OR (IMRangio):ti,ab,kw OR (Microcirculatory Resistance Index Based on Imaging):ti,ab,kw OR (Microvascular Resistance Index Based on Imaging):ti,ab,kw OR (Quantitative flow ratio derived index of microcirculatory resistance):ti,ab,kw OR (Quantitative Microvascular resistance):ti,ab,kw OR (Quantitative microcirculatory resistance):ti,ab,kw OR (quantitative MR):ti,ab,kw OR (quantitative index of Microvascular resistance):ti,ab,kw OR (quantitative index of microcirculatory resistance):ti,ab,kw |
| Embase | ‘angiography-derived index of microcirculatory resistance’:ti,ab,kw OR ‘angiography-derived coronary microcirculatory resistance index’:ti,ab,kw OR ‘angiography-derived index of microvascular resistance’:ti,ab,kw OR ‘angio-index of coronary microcirculatory resistance’:ti,ab,kw OR ‘angio-index of microcirculatory resistance’:ti,ab,kw OR ‘angio-index of microvascular resistance’:ti,ab,kw OR ‘angio-IMR’:ti,ab,kw OR ‘angiography-derived IMR’:ti,ab,kw OR ‘Angio-Based Index of Microcirculatory Resistance’:ti,ab,kw OR ‘Angio-Based Index of microvascular Resistance’:ti,ab,kw OR ‘A-IMR’:ti,ab,kw OR ‘Angio-IMR’:ti,ab,kw OR ‘angiographic microvascular resistance’:ti,ab,kw OR ‘angiographic microcirculatory resistance’:ti,ab,kw OR ‘Angiography-based index of microcirculatory resistance’:ti,ab,kw OR ‘AccuIMR’:ti,ab,kw OR ‘coronary angiography-derived index of microcirculatory resistance’:ti,ab,kw OR ‘coronary angiography-derived index of microvascular resistance’:ti,ab,kw OR ‘CaIMR’:ti,ab,kw OR ‘Index of Microvascular resistance’:ti,ab,kw OR ‘index of microcirculatory resistance’:ti,ab,kw OR ‘IMRangio’:ti,ab,kw OR ‘Microcirculatory Resistance Index Based on Imaging’:ti,ab,kw OR ‘Microvascular Resistance Index Based on Imaging’:ti,ab,kw OR ‘Quantitative flow ratio derived index of microcirculatory resistance’:ti,ab,kw OR ‘Quantitative Microvascular resistance’:ti,ab,kw OR ‘Quantitative microcirculatory resistance’:ti,ab,kw OR ‘quantitative MR’:ti,ab,kw OR ‘quantitative index of Microvascular resistance’:ti,ab,kw OR ‘quantitative index of microcirculatory resistance’:ti,ab,kw |
| Scopus | ABS ((((((((((((((((((((((((((((((angiography-derived index of microcirculatory resistance) OR (angiography-derived coronary microcirculatory resistance index)) OR (angiography-derived index of microvascular resistance)) OR (angio- index of coronary microcirculatory resistance)) OR (angio- index of microcirculatory resistance)) OR (angio- index of microvascular resistance)) OR (angio-IMR)) OR (angiography-derived IMR)) OR (Angio-Based Index of Microcirculatory Resistance)) OR (Angio-Based Index of microvascular Resistance)) OR (A-IMR)) OR (Angio-IMR)) OR (angiographic microvascular resistance)) OR (angiographic microcirculatory resistance)) OR (Angiography-based index of microcirculatory resistance)) OR (AccuIMR)) OR (coronary angiography-derived index of microcirculatory resistance)) OR (coronary angiography-derived index of microvascular resistance)) OR (CaIMR)) OR (Index of Microvascular resistance)) OR (index of microcirculatory resistance)) OR (IMRangio)) OR (Microcirculatory Resistance Index Based on Imaging)) OR (Microvascular Resistance Index Based on Imaging)) OR (Quantitative flow ratio derived index of microcirculatory resistance)) OR (Quantitative Microvascular resistance)) OR (Quantitative microcirculatory resistance)) OR (quantitative index of Microvascular resistance)) OR (quantitative index of microcirculatory resistance)) |
